# Case Report: A complex case of neuropathy and temporomandibular disorders manifesting as otalgia

**DOI:** 10.3389/froh.2025.1595944

**Published:** 2025-09-25

**Authors:** Mayank Shrivastava, Jacob Soliman, Liang Ye

**Affiliations:** 1 Adams School of Dentistry, University of North Carolina, Chapel Hill, NC, United States; 2 Leonard M. Miller School of Medicine, University of Miami, Miami, FL, United States

**Keywords:** temporomandibular disorders, otalgia, nervus intermedius neuropathy, botulinum toxin, pain

## Abstract

Patients with temporomandibular disorders (TMDs) often present with otological symptoms as their primary complaint. Nervus intermedius neuropathy is a rare condition characterized by paroxysmal episodes of sharp, deep ear pain. The presence of overlapping symptoms of TMD and nervus intermedius neuropathy, associated with psychological comorbidities and sleep disturbances, poses significant diagnostic and management challenges for clinicians. Therefore, we report the case of a young female patient who presented to the orofacial pain clinic with debilitating ear pain affecting her quality of life. The patient responded partially to medications and conservative therapies. Adding psychosocial interventions along with botulinum toxin provided notable relief in refractory pain characteristics. In conclusion, although nervus intermedius neuropathy is uncommon in clinical practice, it may coexist with painful TMD and sleep and psychological problems. In such cases of refractory chronic pain, botulinum toxin injections may serve as a critical component for effective patient management.

## Introduction

Orofacial pain is a multidimensional experience that can significantly impact an individual's physical and psychological well-being. Orofacial pain disorders are categorized into distinct groups, including odontogenic, musculoskeletal, neuropathic, headaches, and others. Among these, temporomandibular disorders (TMDs) are a cluster of musculoskeletal conditions affecting the temporomandibular joint (TMJ), the masticatory muscles, and associated anatomical structures (1). TMDs are often linked to psychological distress, sleep problems, and other overlapping pain conditions. It affects approximately 10%–15% of the population at a clinically significant level with symptoms severe enough to necessitate medical attention (2, 3).

Pain is the most common reason individuals seek medical treatment. It may present as either acute or chronic. Painful TMD is characterized as chronic primary pain when no underlying muscle or joint pathology explains the clinical presentation and as chronic secondary pain when an underlying condition, such as rheumatoid arthritis, manifests as TMD. Additional symptoms of TMD include joint noises, restricted jaw movement, cervical pain, and headaches (2). TMDs often coexist with otologic symptoms such as otalgia (earache), tinnitus, aural fullness, and dizziness. Clinically, diagnosing ear pathology or otalgia is particularly challenging due to overlapping innervation and the complex anatomical relationships between the TMJ and other surrounding structures (4, 5).

Barotrauma often results in middle and internal ear injuries, TMD, headaches (migraines and tension-type headaches), sinusitis, and odontogenic pain (barodontalgia). It can occur during activities that involve pressure changes, such as scuba diving, flying, mountain climbing, and hyperbaric oxygen therapy. In some instances, barotrauma can lead to neuropathy (with or without pain) of cranial nerve (CN) V and CN VII. It can also impair special sensory systems, leading to blurred vision, change or loss of taste, hearing loss, and facial palsy (baroparesis) (6, 7).

Previous case reports and epidemiological studies have discussed the association between otological symptoms and TMD secondary to barotrauma (8–12). However, when an individual presents with concurrent symptoms of painful TMD, headaches, otologic complaints, and neuropathy, obtaining a definitive diagnosis becomes challenging. The overlapping symptoms complicate clinical interpretation, increasing the risk of diagnostic errors. Furthermore, when pain is persistent and lacks an identifiable physical or structural etiology, effective diagnosis and management become even more difficult for clinicians.

This case report describes a case of a young female patient referred to an orofacial pain clinic with severe, debilitating right-sided ear pain and TMD precipitated by scuba diving-induced barotrauma. It also aims to familiarize practitioners with conservative treatment approaches performed for managing chronic pain with particular emphasis on the use of botulinum toxin as a treatment option in cases of refractory pain.

## Case report

This case report follows the CARE guidelines (13).

## Clinical history

**Figure 1:**
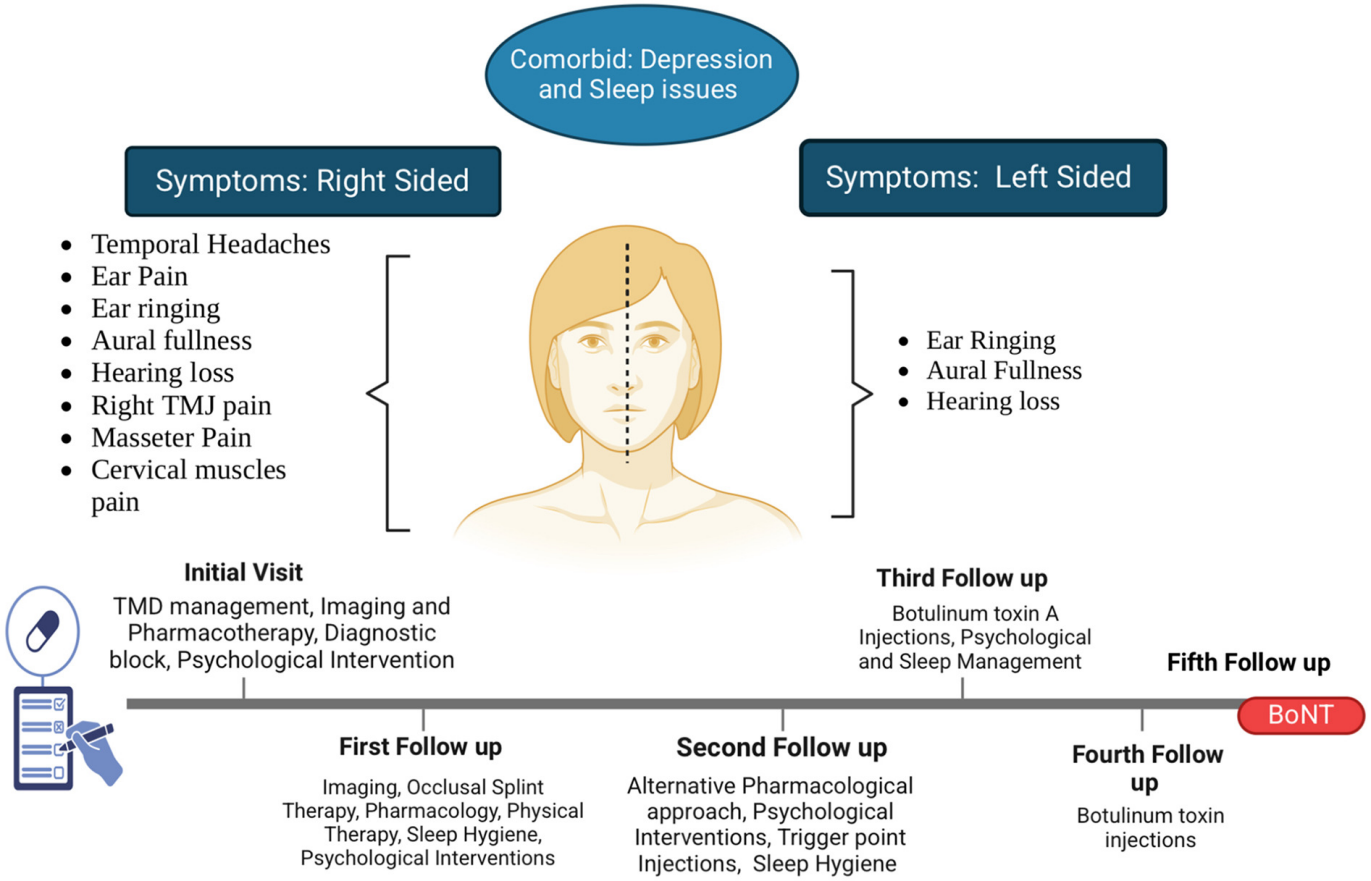
Clinical symptoms comorbidities and timeline for management strategies.

A 19-year-old female patient was referred to the orofacial pain clinic by an otolaryngologist with complaints of right-sided (R) ear pain that began after a scuba diving incident 1 year ago. She described the pain as episodic, sharp, and located deep within the R ear, with a 9–10/10 intensity on the numeric rating scale (NRS, 0 = no pain and 10 = worst possible pain). Initially, the pain occurred 1–2 times a day but had progressively worsened over the past month, increasing to 7–10 episodes daily with each episode lasting a few seconds to 1 min. The pain was spontaneous, disrupted her sleep (causing 1–2 awakenings), and significantly impacted her daily functioning and quality of life, which was the main reason for the consultation. Cold weather, stress, and loud noises were reported as aggravating factors, while no specific alleviating factors were identified. Associated symptoms included tinnitus (ringing in both ears), aural fullness, and bilateral hearing loss (worse in the R ear) (Figure 1).

Additionally, the patient reported a constant dull pain (distinct from the primary ear complaint) on the R side TMJ, masseter, temporalis, and cervical muscles, with an intensity of 4–5/10 on the NRS. Stress exacerbated the pain, whereas sleep and as-needed over-the-counter ibuprofen (800 mg) helped alleviate it. There were no migraine features or autonomic symptoms reported. The patient denied any TMJ noises, jaw locking, limited mouth opening, or occlusal issues. She reported parafunctional habits, such as teeth clenching at night and during the day, and a stiff jaw sensation.

## Medical history

The patient had a medical history of seasonal allergies and depression, managed with cetirizine hydrochloride (antihistamine), sertraline (with an increased dose from 50 to 100 mg daily in the past 3 months), vitamin D, and melatonin. The patient denied any allergies to medications.

## Psychosocial history

The patient is a full-time student and a professional athlete. She was alert and oriented to person, place, and time. She reported an average of 7–8 h of sleep, with a sleep latency of 60–90 min and an average of 1–2 awakenings per night. She reported a persistent feeling of stress and worry, which contributed to difficulty concentrating, academic decline, and withdrawal from sports and other activities due to chronic pain and lack of a precise diagnosis. She denied suicidal ideation and reported no past or current use of alcohol, tobacco, or recreational drugs.

## Previous evaluations

The patient had been evaluated by multiple specialists, including two otolaryngologists (ENTs), a primary care physician, and a dentist. Imaging modalities, including brain magnetic resonance imaging (MRI) with or without contrast, and CT of the temporal bones, revealed no significant findings. Tympanometry revealed mixed conductive sensorineural hearing loss [R ear worse than left (L) ear]. Based on these evaluations, she was initially diagnosed with otalgia secondary to infection, tinnitus, mixed conductive hearing loss, and tonsillitis. She was prescribed non-steroidal anti-inflammatory drugs (NSAIDs), hydrocodone–acetaminophen combination, oral antibiotics, and steroids, but reported no noticeable improvement. The ENT specialists recommended hearing aids, tympanostomy tube placement, and tonsillectomy for management of her conductive hearing loss and recurrent infections.

## Clinical examination

A comprehensive clinical examination of the head and neck regions was conducted. Gross CN I-XII revealed hearing loss (R > L) without motor deficits. Otoscopy showed no signs of inflammation or infection. The external ear and auditory canal were within normal limits. No sharp pain was elicited during palpation of the ear and pre- and post-auricular areas and with the Q-tip test. The Diagnostic Criteria for Temporomandibular Disorders (DC/TMD) clinical examination revealed a maximum mandibular opening of 53 mm and a normal eccentric movement. Palpation of the R TMJ, masticatory muscles (R masseter and R temporalis), and bilateral cervical muscles revealed familiar tenderness, dull pain, and headache without sharp pain to the ear. No TMJ sounds were detected. Mandibular provocation and mandibular function test were normal. Occlusion was normal without odontogenic pathology.

## Investigations

**Figure 2:**
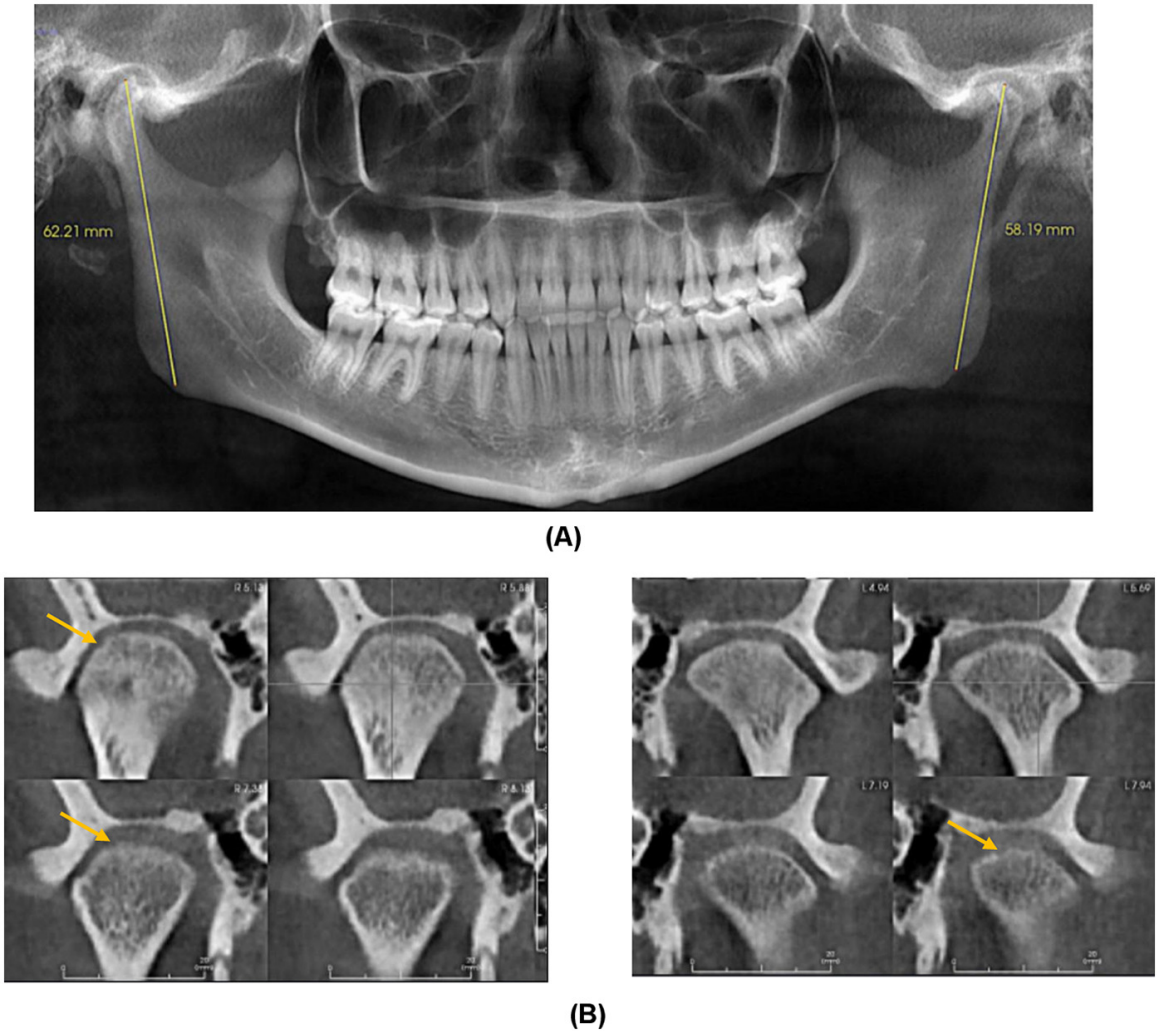
**(A)** Panoramic Image demonstrating increased right ramus height compared to left. **(B)** Coronal Sections of right and left condyle showing mild cortical erosion.

A diagnostic local anesthetic block to the R auriculotemporal nerve and R masseter trigger point resulted in the resolution of dull pain without effect on the sharp ear pain. MRI of the brain with and without contrast, with the R ear as the region of interest, revealed no intracranial or otologic pathology or cranial nerve compression or other structural abnormalities. Cone beam computed tomography (CBCT) imaging revealed mild degenerative changes in both TMJs with increased ramus height on the R side (Figure 2).

Additionally, several screening questionnaires were used before and after adequate pain control: Hospital Anxiety and Depression Scale to quantify anxiety and depression (HADS ≥ 11 for anxiety and depression), Posttraumatic Stress Disorder (PTSD) Checklist—Civilian Version (PCL-C ≥ 36), Pittsburgh Sleep Quality Index (PSQI ≥ 5), Epworth Sleepiness Scale (ESS), ID migraine screener (≥2), and Jaw Functional Limitation Scale (JFLS-8).

After ruling out other pathologies and conducting a thorough examination, the clinical diagnosis included possible nervus intermedius neuropathy (neuropathic pain), R TMJ arthralgia, chronic myofascial pain of masticatory and cervical muscles, headache attributed to TMD, and bilateral TMJ mild degenerative joint diseases. The precipitating factors were barotrauma, with contributing factors including poor sleep, psychosocial stressors, muscle cocontraction, and oral parafunctional habits.

## Management

Treatment recommendations included patient education, pharmacotherapy, home self-care management, a full-coverage intraoral occlusal stabilization appliance, behavioral therapy, physical therapy, and acupuncture. Given the high scores on HADS, PTSD, and PSQI, the patient was referred to a pain psychologist. After discussing the potential interactions and side effects, the patient was prescribed gabapentin 300 mg at bedtime, with instructions to gradually increase the dose by 300 mg every 3 days, provided no side effects were noticed. Communication between the patient and the care team was maintained throughout the titration process. The follow-up timeline is presented in Table 1.

**Table 1:** Timeline of patient complaints, pain characteristics, diagnosis, and treatment.

Timeline	Patient complaint	Pain characteristics	Diagnosis	Treatment
First evaluation	Pain: right ear, TMJ, masticatory muscle, cervical muscle pain, headaches, aural fullness, ringing in the ears, and hearing loss	Constant dull pain (NRS 4–5/10) overlaps with multiple sharp pain episodes (9–10/10)	Possible nervus intermedius neuropathy, right TMJ arthralgia, myalgia of masticatory and cervical muscles, headache attributed to TMD (mixed) conductive sensorineural hearing loss, comorbid with sleep issues and depression	Conservative management (patient education, hot fomentation, massage), occlusal splint therapy Pharmacotherapy (muscle relaxants, NSAIDs, steroids, anticonvulsants) Sleep hygiene, psychological interventions, diagnostic block, and cone beam CT imaging
First follow-up after 1 month since the initial visit	Pain: right ear, TMJ, masticatory muscle, cervical muscle pain, headaches, aural fullness, ringing in the ears, and hearing loss (no improvement in pain)	Constant dull pain (NRS 4–5/10) overlaps with multiple sharp pain episodes (9–10/10)	New diagnosis: mild degenerative disease of bilateral TMJs with right ramus	Continued all the management strategies Pharmacotherapy (anticonvulsants and muscle relaxants) Delivered occlusal splint, physical therapy, and psychological intervention
Second follow-up after 3 months since the initial visit	Pain: right ear, TMJ, masticatory muscle, cervical muscle pain, headaches, aural fullness, ringing in the ears, and hearing loss (mild improvement in pain)	Constant dull pain (NRS 3–4/10) overlaps with multiple sharp pain episodes (8–9/10)		Continue the management strategies, trigger point injections, acupuncture, and neurofeedback
Third follow-up after 6 months since the initial visit	Pain: right ear, TMJ, masticatory muscle, cervical muscle pain, headaches, aural fullness, ringing in the ears (no change in pain characteristics)	Constant dull pain (NRS 3–4/10) overlaps with multiple sharp pain episodes (9/10)	Mixed (conductive sensorineural hearing loss (managed by ENT with hearing aids)	Continue muscle relaxants, terminated anticonvulsant medication due to side effects, continue psychological interventions, and started botulinum toxin injections (100 U)
Fourth follow-up after 9–10 months since the initial visit	Pain: right ear, TMJ, masticatory muscle, cervical muscle pain, headaches, aural fullness (80% improvement), while ringing in the ears persists	Constant dull pain (NRS 1–2/10) and only 3 episodes of sharp pain in the last 3 months (4/10)	Possible nervus intermedius neuropathy, right TMJ arthralgia, myalgia of masticatory and cervical muscles, headache attributed to TMD, comorbid sleep issues, and depression	Continue botulinum toxin therapy, muscle relaxant, and psychological intervention
Fifth follow-up after 1 year since the initial visit	Pain: right ear, TMJ, masticatory muscle, cervical muscle pain, headaches, aural fullness (85% improvement), while ringing in the ears persists	Constant Dull pain (NRS 1–2/10) and only 1 episode of sharp pain in the last 3 months (4/10)	Adequate Pain Control: possible nervus intermedius neuropathy, right TMJ arthralgia, myalgia of masticatory and cervical muscles, headache attributed to TMD, comorbid sleep issues, and depression	Patient agreed to maintain regular follow-up appointments every 4 months and continue the conservative therapy and consultation with a psychologist for the long term if there is no recurrence of pain

Additionally, a low-dose muscle relaxant (cyclobenzaprine 10–20 mg) was prescribed at bedtime. At a gabapentin dose of 2,700 mg, the patient reported mild improvement in the frequency of sharp pain episodes (from 7 to 9 episodes/day to 4–5 episodes per day) without change in duration and or intensity. She also reported a mild reduction in the intensity of constant dull pain. The patient reported improved sleep without nocturnal awakening due to pain; however, she experienced side effects at a higher gabapentin dose, including dizziness, fatigue, weight gain, dry mouth, and blurred vision. Upon patient request, the gabapentin dose was tapered to 900 mg. A trial of oxcarbazepine and baclofen was also attempted over 3 months after the baseline bloodwork; however, the patient was unable to tolerate the side effects and hence discontinued both medications.

**Figure 3:**
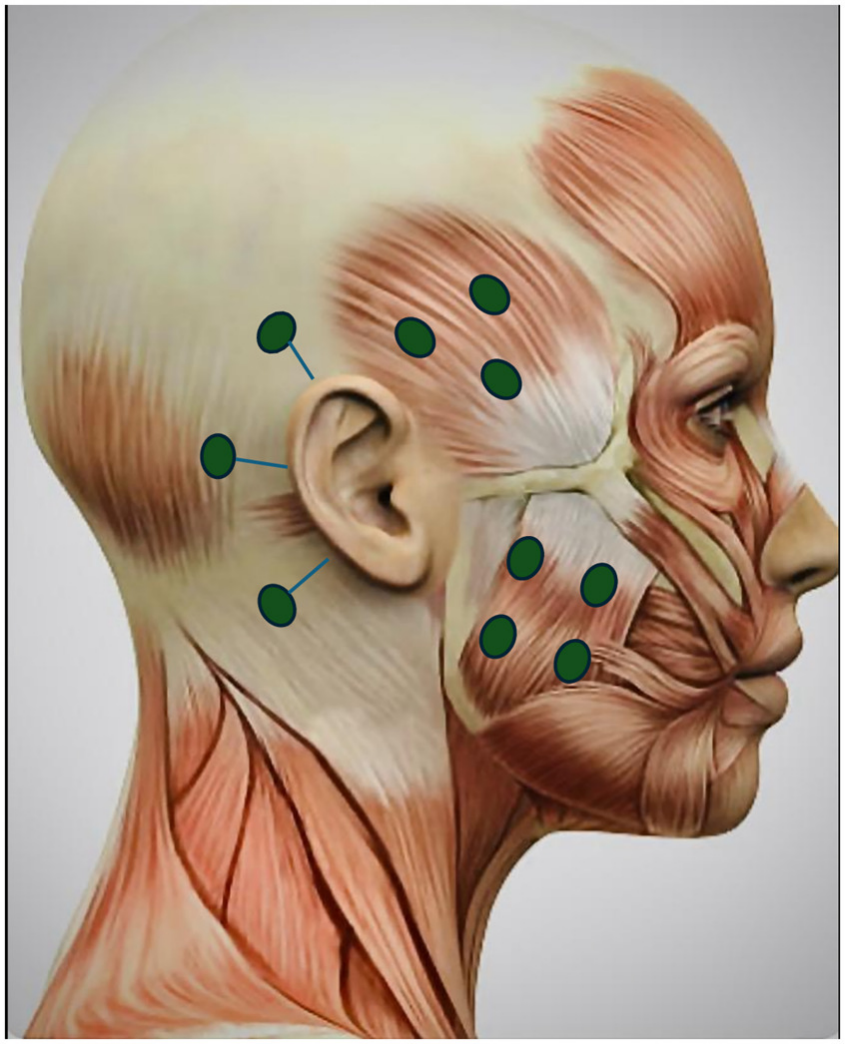
Botulinum toxin injection sites for masseter, temporalis muscles, and three other sites 1 cm from the superior aspect of the auricle, 1 cm behind the auricle, and 1 cm behind the inferior aspect of the auricle (5 U each site bilaterally). Adapted with permission from “Female Head Muscles Anatomy - Side view stock photo” by decade3d, licensed under Standard License.

As the conservative and pharmacological management failed to provide adequate pain relief, a trial of botulinum toxin A injections (100 U) was administered. The injections were administered bilaterally into the masseter (20 U each side) and temporalis (15 U each side) muscles and subcutaneously into the three sites around the ear (5 U each side): 1 cm above superior aspect of the auricle, 1 cm behind the superior aspect of the auricle, and 1 cm behind the inferior aspect of the auricle (Figure 3). Constant communication was maintained with the psychologist regarding therapies such as cognitive behavioral therapy, mindfulness-based therapy, and neurofeedback to address the emotional stressors.

After 3 and 6 months of botulinum toxin injection therapy, the patient reported 85%–90% improvement in pain characteristics with complete resolution of sharp pain episodes. Posttreatment screening questionnaires showed significant improvement (Table 2). The patient agreed to attend regular follow-up appointments every 4 months and to continue the conservative therapy for the long term. Her symptoms improved significantly, and she maintained a normal quality of life.

**Table 2:** Patient-reported outcome measure.

Questionnaire	Screening questionnaire	
	At the initial consultation	After adequate pain relief
Hospital Anxiety and Depression Scale (HADS ≥ 11)	Anxiety = 10 Depression = 16	Anxiety = 2 Depression = 4
Posttraumatic Stress Disorder Checklist—Civilian Version (PCL-C ≥ 36)	Score = 67	Score = 27
Epworth Sleepiness Scale (ESS)	Score = 10	Score = 6
Pittsburgh Sleep Quality Index (PSQI ≥ 5)	Score = 10	Score = 4
ID migraine screener (≥ 2)	Score = 1	Score = 0
Jaw Functional Limitation Scale (JFLS-8)	Score = 23	Score = 12

## Discussion

This case report highlights the complexity of diagnosing and managing debilitating otalgia occurring secondary to nervus intermedius neuropathy and painful TMD. Typically, otalgia can occur from primary ear conditions such as ear infections or can arise from pathological processes and structures other than the ear, called secondary or referred otalgia. Common secondary causes of otalgia include neuralgia or neuropathy, temporomandibular disorders, carotidynia, cervical causes, lesions or tumors, Bell's palsy, Ramsay Hunt syndrome, and others. Otalgia from neuropathy is rare in clinical practice and can be triggered by irritation of any of the sensory nerves. The sensory innervation of the ear is intricate, for instance, the auricle and peri-auricular tissues are innervated by CN V, VII, and X and cervical nerves C1, C2 and C3; the external auditory meatus and canal by CN V, VII, and X; the tympanic membrane by CN VII, IX, and X; and the middle ear by CN V, VII, and IX (14).

Previous studies indicate that middle ear barotrauma is the most common form of barotrauma and typically resolves soon without any serious complications (6, 12, 14, 15). However, inner ear involvement is less common but often severe, potentially resulting in permanent hearing loss, as observed in this case (15). Generally, the characteristics of pain symptoms can provide important diagnostic clues. For example, pain associated with infection is usually continuous and resolves completely within a few days or weeks (16). In this case, the patient identified scuba diving as a precipitating factor for the debilitating sharp pain. An unusual presentation of barotrauma, which serves as the primary diagnosis in this case, is episodic, paroxysmal, sharp ear pain referred to as nervus intermedius neuropathy. Sensory nerves are vulnerable to mechanical pressure or trauma with most cases categorized as facial nerve or trigeminal barotrauma depending on the involvement of a particular nerve. The nervus intermedius is a small branch of the facial nerve that contains both parasympathetic and sensory fibers. When affected, it can cause severe, intense neuralgic pain (17). According to the International Classification of Headache Disorders (ICHD-3), a diagnosis of nervus intermedius neuralgia is established based on paroxysmal attacks of unilateral pain within the nerve distribution, fulfilling specific pain characteristics. These include pain lasting from a few seconds to minutes, severe in intensity, sharp shooting pain, and precipitation by stimulation of a trigger area in the posterior wall of the auditory canal and or preauricular region (18).

The ICHD-3 diagnosis requires the presence of a trigger zone, which is often reported in the literature as localized to the external ear. However, in this case, no trigger zone was identified similar to the previous supporting literature in which one-third of the reported cases lack a definite trigger or non-tactile triggers, leading to the use of the term “possible” nervus intermedius neuralgia (17–19). Multiple cases of nervus intermedius neuropathy have been documented across all age groups with a mean age of onset of 45 years, and only a limited number of cases have been reported in adolescents and pediatric patients. The literature reported a slight predominance of females compared with males similar to our case. Interestingly, previous cases’ clinical presentations vary from ICHD-3 criteria as well. In a review of published cases, 69% of cases met the criteria with classic paroxysmal symptoms, 30% reported dull or aching pain, and 21.7% described a burning sensation or a combination of paroxysmal and dull pain (17, 18, 20). In some cases, autonomic symptoms (lacrimation) were also reported, which is not a typical feature in our case. Nervus intermedius neuropathy can coexist with other neuralgias, such as trigeminal, glossopharyngeal, and occipital neuralgia. Many cases have been documented with concomitant features of other neuralgias (17, 18). However, this report lacks the clinical evidence of other neuralgias and associated pain conditions. For instance, the presence of a trigger zone confined to the trigeminal, glossopharyngeal, and occipital region and sharp pain referral to the face were not present. Furthermore, the diagnosis of auriculotemporal neuralgia was not supported because there was no cessation of pain following an anesthetic block. Moreover, we do not have specific criteria individually mentioned in the ICHD-3 to guide clinicians on auriculotemporal and greater auricular neuralgia. Imaging is typically useful in determining the etiology, and previous cases have reported neurovascular conflict and been managed with surgery. Additionally, most published cases have demonstrated a neurovascular conflict, predominantly involving the anterior inferior communicating artery, a finding that contradicts our case. The presence of normal imaging findings ruled out the possibility of lesions, tumors, and carotidynia, further complicating the clinical diagnosis.

Studies have demonstrated that scuba divers frequently reported pain in the masticatory muscles and TMJ as a result of intense pressure (21, 22, 23). In some reports, TMD is also the main source of ear pain, reporting 25%–65% (16, 24–27). In this case, the familiar dull pain was noted over the joint, masticatory, and cervical muscles. The diagnosis of TMD was rendered based on the DC/TMD examination. However, predisposing variables were presumed to be emotional stressors, poor sleep, and parafunctional habits.

Painful TMD continues to be a diagnostic challenge due to complex relations between the signs and symptoms that are or are not related to the disorder. Often coexistence of non-specific symptoms (fullness, ringing in the ears, or tinnitus) with painful TMD can make diagnosis difficult. Additionally, long-lasting undiagnosed pain conditions can increase the prevalence of associated sleep disturbances and psychological comorbidities such as depression, anxiety, pain catastrophizing, or rumination and vice versa (28). In this case, the patient reported greater scores on the questionnaire used to assess the psychosocial and sleep status. These are the key variables that amplify the pain perception and maintain the chronicity of the condition. In addition to physical pain management, the authors’ goal of treatment is to resolve the associated conditions of sleep and psychological comorbidities. Hence, as a clinician, identifying how pain affects sleep or depression and vice versa is likely to yield improved outcomes for patients experiencing chronic pain. Taken together, these clinical findings in the present case strongly imply that all these conditions, pain, depression, and sleep disturbances, are fundamentally interconnected and highlight the need for a multidisciplinary approach, including sleep hygiene, cognitive behavioral therapy, and biofeedback sessions in managing the pain.

The most common effective treatments reported in the literature for nervus intermedius neuropathy were pharmacological management, such as anticonvulsants (carbamazepine, oxcarbazepine, gabapentin, pregabalin), serotonin or epinephrine reuptake inhibitors (duloxetine, venlafaxine), tricyclic antidepressants (amitriptyline), tramadol, opioids, NSAIDs, and surgical procedures. Interestingly, the patient has shown complete remission of symptomatology at her longest follow-up with a combination of surgery and medications (29, 30). The patient received anticonvulsant oxcarbazepine, gabapentin, and baclofen for the recommended period and on higher dosage; however, medications were withdrawn due to side effects and patient non-compliance. For management of painful TMD, prevailing treatment modalities were implemented, including self-care, oral appliances, physical therapy, ergonomic modification, muscle relaxants, mindfulness, sleep hygiene, stress management, and relaxation techniques (28).

The treatment approaches outlined above generally provide adequate pain relief. However, in this case, considerable barriers such as psychological trauma, emotional distress, and sleep disturbances predispose the patient to central sensitization, which is the probable reason the patient was refractory to a conservative approach. In this case report, the patient has a diagnosis of depression as a “pre-existing factor” that made the patient more vulnerable to chronic pain disorder. Similarly, other factors such as trauma and lack of precise diagnosis may have elevated the risk of pain-related disability in the patient (e.g., fear-avoidant behavior or social withdrawal), influencing the pain-related outcome. Hence, psychological intervention was carried out under the presumption that addressing the psychosocial variables, for instance, reducing distress, improving pain coping, will positively affect the quality of life. However, in this case, the patient was centrally sensitized due to prolonged nociceptive input, and pain had been bothersome for the patient for a long time. A trial of botulinum toxin injections along with psychological intervention was performed due to its antinociceptive central effects.

There is evidence that botulinum toxin attenuates pain independently of its paralytic effect at both peripheral and central levels, resulting in potentially effective management of painful TMD and neuropathic pain (31–33). Given the increasing usage of botulinum toxin, a 100 U trial was conducted in the masticatory and cervical muscles and around the ear to maximize the dose. However, consensus on its indication, dosage, and injection sites is still lacking. It has been suggested that in addition to blocking the release of acetylcholine, botulinum toxin may inhibit the release of local nociceptive neuropeptides such as substance P, calcitonin gene-related peptide (CGRP), and glutamate. It also inhibits neurogenic inflammation and peripheral and central mechanisms of sensitization (33, 34). Studies have shown the efficacy of botulinum toxin A in the treatment of TMD-related myofascial pain (35–37). Reports have suggested that toxins when injected in hyperalgesic tissues may help reduce spontaneous and provoked pain of neuropathic origin (33). Given that there are no major side effects from botulinum toxin injections, patients with poorly controlled orofacial pain conditions may benefit from the adjunct use of botulinum toxin injections for reducing the intensity and frequency of triggering.

Therefore, the main strength of this case report is to enhance awareness of the clinical presentation of nervus intermedius neuropathy that does not meet precise diagnostic criteria and coexisting orofacial pain conditions. The case report further emphasized the importance of adopting the standardized DC/TMD criteria and reliable screening tools to identify the underlying psychological and sleep disorders. However, the literature does not provide adequate evidence on the management of nervus intermedius neuropathy, where pain is persistent and devoid of any physical or structural causes. The report highlighted the use of botulinum toxin injections as an adjunct method in the management of such painful conditions. The main limitation is a clear lack of standardized treatment and missing data on appropriate follow-up to determine treatment success. There has not been an adequate trial of alternative medications, and the authors have not provided information on how to address additional non-specific symptoms. It is necessary to highlight that there is insufficient evidence to suggest that the described case may not be actual nervus intermedius neuropathy. As a result, studies with an overlapping clinical presentation of other orofacial pain conditions and different phenotypes of nervus intermedius neuralgia are warranted.

## Conclusion

This case illustrates the intricate relationship between otalgia, neuropathic pain, temporomandibular disorders, and complications possibly associated with barotrauma. The diagnosis of orofacial pain conditions should be considered when patients have been cleared for ear infections or other ear pathologies, specifically in cases with barotrauma. A multidisciplinary approach is needed in case of refractoriness to pain management. Awareness of the clinical presentation of nervus intermedius neuropathy, psychological, and sleep disorders is essential for improving patient outcomes.

## Data Availability

The original contributions presented in the study are included in the article/Supplementary Material, further inquiries can be directed to the corresponding author.
